# Characteristics and outcomes of patients receiving palliative care in the intensive care unit: retrospective single-center cohort study

**DOI:** 10.31744/einstein_journal/2025AO1491

**Published:** 2025-11-07

**Authors:** Mayara Laíse Assis, Thais Dias Midega, Fábio Tanzillo Moreira, Vinícius Barbosa Galindo, Farah Christina de La Cruz Scarin, Pedro Guadix Zulian Teixeira, Ricardo Luiz Cordioli, Thiago Domingos Corrêa

**Affiliations:** 1 Hospital Israelita Albert Einstein São Paulo SP Brazil Hospital Israelita Albert Einstein, São Paulo, SP, Brazil.

**Keywords:** Palliative care, Patient care team, Professional-family relations, Family support, Attitude of health personnel, Dehumanization

## Abstract

**Objectives:**

To describe the use of advanced life support, resource utilization, and clinical outcomes and to identify the risk factors for late initiation of palliative care support in patients at the intensive care unit.

**Methods:**

A retrospective, single-center cohort study conducted in a private intensive care unit. All patients aged ≥18 years in palliative care admitted to the intensive care unit between January 1, 2014, and December 31, 2018, were eligible. Patients were categorized based on the timing of palliative care initiation: Early Group (palliative care instituted before or within 24 h of intensive care unit admission) and Late Group (palliative care instituted after 24 h of intensive care unit admission). Comparisons were made between the two groups.

**Results:**

Overall, 502 patients were included in this analysis. The Early Group accounted for 37.6% (189/502) of patients and the Late Group for 62.4% (313/502). Patients in the Early Group were older, had a higher modified frailty index, and had lower rates of immunosuppression or hematologic cancers. During the intensive care unit stay, the Early Group required less mechanical ventilation, vasopressors, renal replacement therapy, red blood cell transfusions and parenteral nutrition compared to the Late Group. Additionally, the Early Group had lower intensive care unit and hospital stay and mortality compared to the Late Group. Independent predictors of late initiation of palliative care in the multivariate analysis included younger age, being a surgical patient, chronic kidney disease requiring renal replacement therapy, hematologic cancer, and the need for mechanical ventilation at intensive care unit admission.

**Conclusion:**

Early implementation of palliative care was associated with reduced resource utilization and improved clinical outcomes.

## In Brief

The management of patients receiving palliative care in the intensive care unit remains challenging. Assis et al. compare characteristics and outcomes of patients with early versus late initiation of palliative care, including advanced life support use, mortality, and length of stay.

## Highlights

■ Patients who received early palliative care were younger with more immunosuppression.
■ Intensive care unit and hospital mortality were higher with late palliative care.
■ Early palliative care reduced the use of advanced life support resources.

## Graphical abstract

**Figure:**
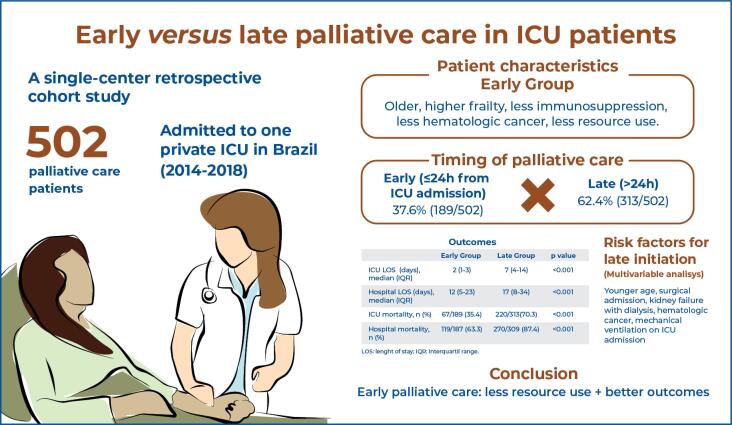

## INTRODUCTION

Technological advancements in intensive care medicine, has provided advanced life support for critically ill patients.^(1,2)^ However, in the final decades of the 20th century, as the global population aged and the prevalence of incurable chronic-degenerative diseases increased, over 70% of deaths occurred in hospitals, specifically in intensive care units (ICUs).^(3)^

In approximately 40% of cases, death in the ICU is preceded by decisions to suspend or stop treatments which are considered futile or excessively obstinate.^(4)^ Therapeutic obstinacy and/or dysthanasia - defined as the use of artificial organ support to prolong life without quality of life and dignity^(5)^ - can impose profound physical, mental, and spiritual suffering on patients, their families, and the multidisciplinary team working in the ICU.^(2)^ Therefore, palliative care is a crucial field in medicine and healthcare, offering relief and dignity to patients with serious and incurable illnesses.^(6,7)^

The World Health Organization (WHO) defines palliative care as an approach that enhances the quality of life of patients facing life-threatening illnesses and their families by preventing and alleviating physical, psychosocial, and spiritual suffering.^(8)^ This definition underscores the importance of shifting the therapeutic focus for patients with terminal illnesses, avoiding unnecessary, ineffective, and painful prolongation of life while ensuring a dignified death.^(9,10)^ Nevertheless, the timely identification of patients who could benefit from palliative care remains challenging. In the hospital setting, such recognition is often delayed, especially when invasive interventions intended to maintain or restore organ function are no longer appropriate or aligned with patient's clinical condition.^(11)^

We hypothesized that the delayed adoption of palliative care for patients with critical illnesses admitted to the ICU is associated with unnecessary exposure to interventions and life-sustaining therapies, resulting in increased resource utilization and longer hospital stays.

## OBJECTIVE

To describe the use of advanced life support, resource utilization, and to report the clinical outcomes of patients in the intensive care units who received palliative care, and to identify the risk factors for the late initiation of palliative care support in this patient sample.

## METHODS

### Study design

We conducted a retrospective, single-center cohort study. The study was approved by the local Ethics Committee of *Hospital Israelita Albert Einstein* (CAAE: 69973717.4.0000.0071; # 3.290.740) and the need for informed consent was waived. This study was reported following the Strengthening the Reporting of Observational Studies in Epidemiology (STROBE) statement.^(12)^

### Setting

This study was conducted in a private quaternary care hospital located in São Paulo, Brazil. The Hospital has 724 inpatient beds, 54 of which were allocated to an open medical-surgical adult ICU.

### Study participants

Adult patients (≥18 years old) who were receiving palliative care at the time of admission to the ICU, and those who had palliative care initiated during their ICU stay between January 1, 2014, and December 31, 2018, were eligible for inclusion in this study.

### Data collection

All study data were extracted from the Epimed Monitor System® platform (Epimed Solutions, Rio de Janeiro, Brazil),^(13)^ a structured electronic tool in which patient data are prospectively entered by trained professionals. The data were extracted, anonymized, and decoded before given to the study's principal investigator by an independent research assistant who was not involved in this project.

### Study variables and follow-up

The collected variables included demographic data, comorbidities, source of ICU admission, category and specific diagnoses at ICU admission, Simplified Acute Physiology Score (SAPS 3) on ICU admission,^(14)^ Charlson Comorbidity Index (CCI),^(15)^ Modified Frailty Index (MFI),^(16)^ reason for ICU admission, use of organ support therapies and resources [vasopressors, non-invasive ventilation (NIV), invasive mechanical ventilation (MV), renal replacement therapy (RRT), parenteral nutrition (PN), and blood component transfusion] within the first hour of ICU admission and throughout the ICU stay, frequency of ICU readmission, ICU and hospital length of stay (LOS)], discharge destination after ICU and hospital discharge, and ICU and hospital mortality. Patients were followed until hospital discharge.

### Definitions

Patients were classified as receiving palliative care if at least one of the following directives was documented in their electronic medical records: "do not resuscitate," "do not dialyze," "do not intubate," or expressions such as "limitation of therapeutic support," "partial palliative care," or "exclusive palliative care." These expressions are commonly used in clinical practice to indicate the adoption of a palliative care approach.

Patients receiving palliative care were classified into two groups based on the timing of palliative care initiation: the Early Group (palliative care implemented before or within the first 24 h of ICU admission) and the Late Group (palliative care implemented after 24 h of ICU admission). At the institution where the study was conducted, the decision to initiate palliative care involves careful deliberation among the attending physician, ICU medical team, palliative care specialist, family members, and when possible, the patient.

### Outcomes

The outcomes of interest included the use of advanced life support (vasopressors, NIV, MV, RRT, PN, and blood component transfusion), resource use (ICU and hospital LOS), and clinical outcomes (ICU and hospital mortality). Exposure was defined by the timing of palliative care initiation (Early Group *versus* Late Group).

### Statistical analysis

Categorical variables are reported as absolute and relative frequencies. Continuous numerical variables are presented as median with interquartile range (IQR). Normality was assessed using the Kolmogorov-Smirnov test.

Comparisons were made between the early and Late Groups. Categorical variables were compared using the χ^2^ test or Fisher's exact test, as appropriate. Continuous variables were compared using the independent *t*-test or the Mann-Whitney U test in cases of non-normal distribution.

Univariable logistic regression analysis was performed to address risk factors associated with delayed initiation of palliative care (*i.e*., initiation after 24 h of ICU admission - Late Group). Multivariable logistic regression analyses, using a backward elimination procedure and including all the predictors showing a p<0.20 in the univariable analysis, were conducted to obtain adjusted odds ratio (OR) along with a 95% confidence interval (95%CI) to identify which predictors were independently associated with late initiation of palliative care. The final model included only variables significantly associated with delayed initiation of palliative care.

Collinearity was assessed using the variance inflation factor (VIF), with a VIF >2.5 arbitrarily defined as indicative of collinearity. The linearity assumption for continuous variables included in logistic regression models was tested by analyzing the interaction between each predictor and its natural log transformation.^(17)^ When the linearity assumption was violated, continuous numerical variables were categorized.^(17)^ The final multivariable logistic regression model discrimination, as measured by the area under a receiver operating characteristic curve (AUC), and calibration, as assessed by the Hosmer-Lemeshow chi-square statistic, were reported.^(18)^

Two-tailed tests were used, with statistical significance set at p<0.05. All analyses were conducted using IBM (SPSS) Statistics for Macintosh, version 29 (IBM Corp., Armonk, NY, USA).

## RESULTS

### Patients

**Figure 1:**
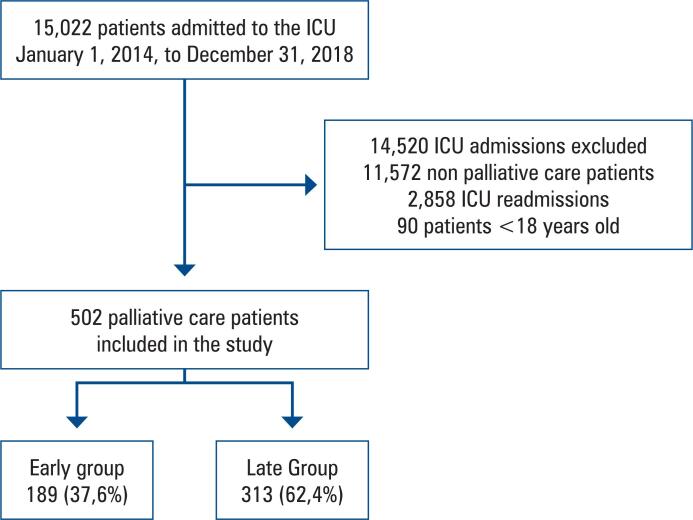
Study flow chart

Between January 1, 2014, and December 31, 2018, 15,022 patients were admitted to the ICU (Figure 1). After excluding 14,520 patients admitted to the ICU, 502 patients were included in the final analysis (Figure 1). Palliative care was initiated before or within 24 h of ICU admission in 37.6% (189/502) of patients (Early Group) and after 24 h of ICU admission in 62.4% (313/502) of patients (Late Group) (Figure 1).

ICU: intensive care unit.

The baseline characteristics of the included patients are presented in table 1. The median age (IQR) was 83 (70-88) years, with 50.6% of patients being female. The median SAPS 3 score was 63 (54-72), the CCI was 2 (1-4), and the MFI was 3 (2-4) points (Table 1). Most patients (89.8%) were admitted to the ICU due to a medical (clinical) condition, primarily from the emergency department (40.6%), with a median (IQR) hospital stay before ICU admission of 1 (0-8) day (Table 1).

**Table 1:** Baseline characteristics of patients

Characteristics		All patients 502 (100%)	Early Group 189 (37.6%)	Late Group 313 (62.4%)	p value*
Age, years (median, IQR)		83 (70-88)	86 (79-92)	78 (64-86)	<0.001#
Female, n (%)		254/502 (50.6)	101/189 (53.4)	153/313 (48.9)	0.370&
SAPS 3 score (median, IQR)		63 (54-72)	64 (57-71)	62 (53-73)	0.232#
Charlson Comorbidity Index		2 (1-4)	2 (1-4)	2 (1-4)	0.721£
MFI points (median, IQR)		3 (2-4)	3 (2-4)	2 (1-3)	<0.001£
MFI frail patient, n (%)		119/502 (23.7)	58/189 (30.5)	62/313 (19.8)	0.009&
Hospital LOS before ICU admission (days), median (IQR)		1 (0-8)	1 (0-7)	1 (0-9)	0.238£
Admission diagnostic category, n (%)					0.019&
	Medical	451/502 (89.8)	179/189 (94.7)	272/313 (86.9)	
	Elective Surgery	28/502 (5.6)	6/189 (3.2)	22/313 (7.0)	
	Emergency surgery	23/502 (4.6)	4/189 (2.1)	19/313 (6.1)	
Source of ICU admission, n (%)					0.020&
	Emergency department	204/502 (40.6)	94/189 (49.7)	110/312 (35.1)	
	Step-down unit	106/502 (21.1)	37/189 (19.6)	69/312 (22.0)	
	Ward	102/502 (20.3)	35/189 (18.5)	67/312 (21.4)	
	Other$	53/502 (10.6)	15/189 (7.8)	38/312 (12.2)	
	Operating room/procedure unit	37/502 (7.4)	8/189 (4.2)	29/312 (9.3)	
Underlying disease, n (%)					
	Systemic hypertension	252/502 (50.2)	103/189 (54.5)	149/313 (47.6)	0.160&
	Cancer	109/502 (21.7)	42/189 (22.2)	67/313 (21.4)	0.918&
	Congestive heart failure	92/502 (18.3)	37/189 (19.6)	55/313 (17.6)	0.657&
	Immunosuppression	91/502 (18.1)	25/189 (13.2)	66/313 (21.1)	0.036&
	COPD	80/502 (15.9)	26/189 (13.8)	54/313 (17.3)	0.362&
	Chronic kidney disease	68 /502 (13.5)	22/189 (11.6)	46/313 (14.7)	0.404&
	Metastatic cancer	58/502 (11.6)	20/189 (10.6)	38/313 (12.1)	0.685&
	Hematologic cancer	42/502 (8.4)	4/189 (2.1)	38/313 (12.1)	<0.001&
	Chronic kidney disease RRT	22/502 (4.4)	5/189 (2.6)	17/313 (5.4)	0.206&
	Liver cirrhosis	20/502 (4.0)	3/189 (1.6)	17/313 (5.4)	0.058&
	Asthma	11/502 (2.2)	3/189 (1.6)	8/313 (2.6)	0.686&
	Complicated diabetes mellitus	4/502 (0.8)	2/189 (1.1)	2/313 (0.6)	1.000&
ICU admission diagnoses, n (%)					0.068&
	Non-operative diagnoses				
	Sepsis	261/502 (52.0)	115/189 (60.8)	146/313 (46.6)	
	Cardiovascular	59/502 (11.8)	23/189 (12.2)	36/313 (11.5)	
	Neurologic	56/502 (11.2)	15/189 (7.9)	41/313 (13.1)	
	Respiratory	40/502 (8.0)	14/189 (7.4)	26/313 (8.3)	
	Gastrointestinal	22/502 (4.4)	9/189 (4.8)	13/313 (4.2)	
	Other	5/502 (1.0)	0/189 (0.0)	5/313 (1.6)	
	Renal	4/502 (0.8)	1/189 (0.5)	3/313 (1.0)	
	Trauma	4/502 (0.8)	2/189 (1.1)	2/313 (0.6)	
	Hematologic	1/502 (0.2)	0/189 (0.0)	1/313 (0.3)	
Operative diagnoses					
	Cardiovascular surgery	17/502 (3.4)	5/189 (2.6)	12/313 (3.8)	
	Other	11/502 (2.4)	1/189 (0.5)	10/313 (3.2)	
	Gastrointestinal surgery	10/502 (2.0)	2/189 (1.1)	8/313 (2.6)	
	Neurologic surgery	6/502 (1.2)	0/189 (0.0)	6/313 (1.9)	
	Orthopedic surgery	4/502 (0.8)	2/189 (1.1)	2/313 (0.6)	
	Respiratory surgery	2/502 (0.4)	0/189 (0.0)	2/313 (0.6)	

Data presented as median [interquartile range (IQR)] or n/total n (%).

* p values were calculated using

(#) the independent *t*-test,

(&) the *χ*^2^ test or

(£) the Mann-Whitney U test;

$ Another hospital, another ICU, hospice, and home care.

SAPS 3: Simplified Acute Physiology Score 3; MFI: Modified Frailty Index; LOS: Length of stay; RRT: renal replacement therapy, COPD: chronic obstructive pulmonary disease; ICU: intensive care unit.

Compared to the Late Group, patients in the Early Group were significantly older [86 (79--92) *versus* 78 (64-86) years; p<0.001], had higher MFI [3 (2-4) *versus*. 2 (1-3) points; p<0.001], were more frequently classified as frail (30.5% *versus* 19.8%; p=0.009), and had a lower prevalence of immunosuppression (13.2% *versus* 21.1%; p=0.036) and hematological cancer (2.1% *versus* 12.1%; p<0.001) (Table 1).

### Use of advanced life support

Patients in the Early Group were less likely to receive MV upon ICU admission compared to those in the Late Groups (19.0% *versus* 31.6%; p=0.003), while the use of vasopressors, NIV, and RRT did not differ significantly between the groups (Table 2). During the ICU stay, patients in the Early Group received the following interventions less frequently: vasopressors (45.0% *versus* 71.2%; p<0.001, MV (30.2% *versus* 74.4%; p<0.001), RRT (6.9% *versus* 30.7%; p<0.001), red blood cell transfusion (14.3% *versus* 43.5%; p<0.001), platelets transfusion (4.2% *versus* 18.8%; p<0.001), plasma transfusion (1.1% *versus* 5.8%; p=0.018) and parenteral nutrition (2.6% *versus* 12.5%; p<0.001) (Table 2).

**Table 2:** Advanced Life Support, resource utilization and clinical outcomes

Characteristics		All patients 502 (100%)	Early Group 189 (37.6%)	Late Group 313 (62.4%)	p value*
Support at ICU admission, n (%)					
	Mechanical ventilation	135/502 (26.9)	36/189 (19.0)	99/313 (31.6)	0.003‡
	Vasopressors	133/502 (26.5)	42/189 (22.2)	91/313 (29.1)	0.114‡
	Non-invasive ventilation	129/502 (25.7)	54/189 (28.6)	75/313 (24.0)	0.298‡
	Renal replacement therapy	6/502 (1.2)	1/189 (0.5)	5/313 (1.6)	0.520‡
Support during ICU stay, n (%)					
	Vasopressors	308/502 (61.4)	85/189 (45.0)	223/313 (71.2)	<0.001‡
	Mechanical ventilation	290/502 (57.8)	57/189 (30.2)	233/313 (74.4)	<0.001‡
	Noninvasive ventilation	220/502 (43.8)	84/189 (44.4)	136/313 (43.5)	0.901‡
	Red blood cells transfusion	163/502 (32.5)	27/189 (14.3)	136/313 (43.5)	<0.001‡
	Renal replacement therapy	109/502 (21.7)	13/189 (6.9)	96/313 (30.7)	<0.001‡
	Platelets transfusion	67/502 (13.3)	8/189 (4.2)	59/313 (18.8)	<0.001‡
	Parenteral nutrition	44/502 (8.8)	5/189 (2.6)	39/313 (12.5)	<0.001‡
	Plasma transfusion	20/502 (4.0)	2/189 (1.1)	18/313 (5.8)	0.018‡
ICU LOS (days), median (IQR)		5 (2–10)	2 (1–3)	7 (4–14)	<0.001#
Hospital LOS (days), median (IQR)		15 (7–31)	12 (5–23)	17 (8–34)	<0.001#
ICU readmission at any time		63/502 (12.5)	26/189 (13.8)	37/313 (11.8)	0.620‡
ICU readmission within 24 h		1/502 (0.2)	1/189 (0.5)	0/313 (0.0)	0.799‡
Destination at ICU discharge, n (%)					<0.001‡
	Death (ICU mortality)	287/502 (57.2)	67/189 (35.4)	220/313 (70.3)	
	Step-down unit	175/502 (34.9)	100/189 (52.9)	75/313 (24.0)	
	Floor	26/502 (5.2)	15/189 (7.9)	11/313 (3.5)	
	Another hospital	8/502 (1.6)	3/189 (1.6)	5/313 (1.6)	
	Another ICU	6/502 (1.2)	4/189 (2.1)	2/313 (0.6)	
Destination at hospital discharge, n (%)					<0.001‡
	Death (hospital mortality)	389/469 (78.4)	119/187 (63.6)	270/309 (87.4)	
	Home	96/469 (19.4)	63/187 (34.7)	33/309 (10.7)	
	Another hospital	10/469 (2.0)	4/187 (2.1)	6/309 (1.9)	
	Home care	1/469 (0.2)	1/187 (0.5)	0/309 (0.0)	

Data presented as median [interquartile range (IQR)] or n/total n (%).

* p values were calculated using

(‡) the *χ*^2^ test or

(#) the Mann-Whitney U test.

ICU: Intensive care unit; LOS: length of stay.

### Resource use and clinical outcomes

The Early Group had shorter ICU [2 (1-3) *versus* 7 (4-14) days; p<0.001] and hospital [12 (5-23) *versus* 17 (8-34) days; p<0.001] lengths of stay compared to the Late group (Table 2). ICU mortality (35.4% *versus* 70.3%; p<0.001) and hospital mortality (63.6% *versus* 87.4%; p<0.001) were also lower in the Early Group (Table 2). The frequency of ICU readmission did not differ between the two groups (Table 2).

### Predictors of late palliative care initiation

The results of the univariate and multivariate logistic regression analyses are presented in Table 3. In the multivariate analysis, independent predictors of late initiation of palliative care included younger age, being a surgical patients, presence of chronic kidney disease requiring RRT, hematologic cancer, and need for MV within 1 h of ICU admission (Table 3).

**Table 3:** Univariable and multivariate logistic regression analysis addressing risk factors for late initiation of palliative care

Predictors		Univariable Analysis		Multivariable Analysis	
		OR (95%CI)	p value	OR (95%CI)	p value
Age, years*					
	≥89	1 (Reference)		1 (Reference)	
	83-88	2.08 (1.26-3.43)	0.004	1.96 (1.17-3.28)	0.001
	70-82	2.97 (1.77-4.96)	<0.001	2.44 (1.42-4.21)	0.001
	≤69	6.35 (3.57-11.30)	<0.001	5.67 (3.13-10.27)	<0.001
SAPS 3 score*					
	≤53	1 (Reference)			
	54-63	0.45 (0.26-0.77)	0.003		
	64-72	0.40 (0.23-0.69)	<0.001		
	≥73	0.67 (0.38-1.17)	0.157		
MFI (Points)		0.78 (0.68-0.89)	<0.001		
LOS prior unit admission		0.99 (0.99-1.00)	0.291		
ICU admission diagnostic category					
	Nonoperative (Medical)	1 (Reference)		1 (Reference)	
	Operative	2.70 (1.32-5.52)	0.007	2.35 (1.10-5.02)	0.028
Source of ICU admission					
	Ward	1 (Reference)			
	Step-down unit	0.97 (0.55-1.73)	0.929		
	Emergency department	0.61 (0.37-1.00)	0.050		
	Operating room/procedure unit	2.09 (0.90-4.83)	0.084		
	Others$	1.12 (0.53-2.38)	0.770		
Underlying disease					
	Systemic hypertension	0.76 (0.53-1.09)	0.135		
	Chronic kidney disease RRT	2.11 (0.77-5.83)	0.148	3.15 (1.09-9.10)	0.034
	Immunosuppression	1.75 (1.06-2.89)	0.028		
	Hematologic cancer	6.39 (2.24-18.20)	<0.001	5.42 (1.85-15.85)	0.002
	Liver cirrhosis	3.56 (1.03-12.32)	0.045		
Support at ICU admission					
	Mechanical ventilation	1.97 (1.27-3.04)	0.002	2.03 (1.28-3.23)	0.003
	Vasopressors	1.44 (0.94-2.19)	0.093		

$ Another hospital, another ICU, hospice, and home care;

* SAPS 3=Simplified Acute Physiology Score 3. SAPS 3 score was categorized according to percentiles because the assumption of linearity was violated.

The multivariable model (n=502 patients) had an area under the Receiver Operating Characteristic curve (95%CI) of 0.72 (0.67–0.76) and a Hosmer-Lemeshow *χ*^2^ of 10,505 (p=0.162).

OR: odds ratio; 95%CI: 95% confidence interval; MFI: modified frailty index; LOS: length of stay, ICU: intensive care unit, RRT: renal replacement therapy.

## DISCUSSION

In this retrospective cohort study involving approximately 500 patients with critical illnesses receiving palliative care, we found that those who received palliative care before or within the first 24 h of ICU admission required less invasive organ support, utilized fewer resources, had shorter ICU and hospital lengths of stay, and exhibited lower hospital mortality compared to those who received palliative care after 24 h.

Previous studies have demonstrated that the adoption of palliative care for patients with chronic diseases or progressive, terminal clinical conditions is associated with improved quality of life and a reduction in the frequency and severity of physical symptoms, including pain, dyspnea, fatigue, gastrointestinal symptoms, delirium, anxiety, and depression.^(19,20)^ Consistent with the findings of the present study, early adoption of palliative care has also been associated with reduced use of advanced life support,^(21–23)^ as well as shorter ICU and hospital stays.^(21,24)^ This approach aligns with patients’ preferences and goals, ensuring more personalized care tailored to individual needs and providing greater opportunity for interaction with family members.^(25,26)^ Additionally, it fosters more effective communication among patients, family members, and healthcare professionals, enabling discussions that align treatment decisions with patient values and goals.^(27,28)^

Similar findings were recently reported in a meta-analysis involving 28 studies and 13,664 patients;^(29)^ compared to usual care, the integration of palliative care was associated with reduced utilization of emergency services, lower hospitalization rates, and fewer symptoms in patients with non-oncological chronic diseases.^(29)^ Notably, the implementation of palliative care has also been linked with improved quality of life and reductions in post-traumatic stress disorder, anxiety, and depression in patients with critical illnesses. Temel et al.,^(30)^ in a study involving 151 patients with metastatic lung cancer, demonstrated that initiation of early palliative care, alongside standard oncologic treatment, resulted in better quality of life and a lower incidence of depressive symptoms, compared to the group of patients receiving standard treatment.^(30)^

Despite the documented benefits, several studies conducted in different countries have shown that palliative care remains underutilized or is often initiated late in potentially life-limiting illnesses, such as cancer.^(31–34)^ Multiple factors contribute to the non-adoption or delayed initiation of palliative care in eligible patients. These include limited understanding of the palliative approach among healthcare providers, family-related concerns, cultural and religious differences, ineffective communication among the multidisciplinary team, family, and patients, limited access to specialized palliative care services, and the inherent difficulty in addressing an unfavorable prognosis.^(35–37)^

Significant disparities persist across Latin American countries regarding oncological care, which is also reflected in end-of-life care. Challenges include a lack of advanced care planning, limited access to adequate palliative care, and shortages of pain relief medications.^(38)^ It should be noted that there is greater resistance to referring patients with non-cancer chronic diseases for palliative care, as evidenced in the study by Kawashima et al.^(39)^ Furthermore, the decision-making process regarding the suspension or withdrawal of life-sustaining treatments for patients in the ICU is not uniform worldwide. It is influenced by various factors, including legal, political, and religious considerations, the experience of healthcare professionals, and the individual characteristics of each patient.^(4,40–44)^

Several studies have examined the risk factors or predictors associated with the late initiation of palliative care. Family hesitation, challenges in defining patient prognosis, physician uncertainty, and reluctance among intensivists have been described as key contributors to delayed palliative care initiation.^(10)^ In another study, 1,256 healthcare professionals responded to a questionnaire listing the main barriers to implementing palliative care for patients with critical illnesses in thirteen Canadian university hospitals.^(45)^ The most frequently cited factors include difficulty among patients and/or family members in accepting a poor prognosis, limited understanding of disease complications, and disagreement among family members regarding treatment goals.^(45)^

This study has some limitations. Firstly, the definition of palliative care was imprecise. It encompassed a broad range of situations, from patients with specific limitations (e.g., a directive not to initiate RRT) to those receiving exclusive palliative care. Secondly, because data regarding the limitations of life-sustaining therapies were not collected, we were unable to analyze the nature of these limitations or the timing of their implementation in the course of patients’ illness. Thirdly, the classification of early versus late adoption of palliative care was arbitrary, as there is no universally accepted definition in the literature to distinguish between them. Finally, as an observational study conducted at a single center, the external validity of our findings is limited.

## CONCLUSION

Early implementation of palliative care was associated with reduced resource utilization and improved clinical outcomes. This association suggests that the implementation of palliative care may benefit patients by providing a more holistic and compassionate approach to treatment, while also contributing to more efficient use of healthcare resources.
